# Study on Meso-Structure Evolution in Granular Matters Based on the Contact Loop Recognition and Determination Technique

**DOI:** 10.3390/ma14216542

**Published:** 2021-10-31

**Authors:** Jiake Yang, Qun Qi

**Affiliations:** School of Civil Engineering, Central South University, Changsha 410075, China; YangJack007@outlook.com

**Keywords:** granular matter, DEM, contact network, meso-structure, macro-mechanics, multivariate model

## Abstract

On the mesoscopic scale, granular matter is tessellated into contact loops by a contact network. The stability of granular matter is highly dependent on the evolution of contact loops, including the number and area evolutions of contact loops with different geometric shapes (which can reflect the mechanical variables in the macroscale). For the features of numerous loops with complex geometry shapes in contact network images, a contact loop recognition and determination technique was developed in this study. Then, numerical biaxial compression tests were performed by the discrete element method (DEM) to investigate how the meso-structural indexes evolve along with the macro-mechanical indexes. The results show that the proposed *Q-Y* algorithm is effective in determining the geometric types of contact loops from contact network images. The evolution of contact loops is most active in the hardening stage, during which the number percentages of *L*_3_ (loops with three sides) and *L*_6+_ (loops with six or more sides) show opposite evolution patterns. For the area percentage, only *L*_6+_ increases while others decrease. Considering the meso-structural indexes (number percentage and area percentage of loops) are sensitive to the change of macro-mechanical indexes (deviatoric stress, axial strain, and volumetric strain) in the hardening stage. Multivariate models were established to build a bridge between the meso-structure and the macro-mechanics.

## 1. Introduction

The analysis of microstructure (such as particle rolling, contact sliding, coordination number, and contact anisotropy, etc.) is helpful to explain the complex macroscopic behaviors (such as strength and deformation characteristics) of granular materials [1,2,3,4,5]. However, establishing the relationship between microscopic variables and macroscopic behavior requires an intermediate scale, namely the mesoscale [6,7]. It has been widely accepted that the geometric arrangement and combination of particles determine the macroscopic mechanical behaviors of particle assembly [8]. At the mesoscale, structural features are accounted for with clusters of a few interacting particles. Therefore, the structure of particle assembly can be divided into elementary “contact loops” (hereinafter referred to as loops), which are enclosed by the contact branches of interacting particles [9]. The stability of assembly is highly dependent on the evolution of contact loops, which can help understand the underlying mechanisms behind macroscopic observations [10].

The edge number of the contact loop plays an important role in the mechanical behavior of assembly. For example, the increase in edge number will increase the freedom degree of contact loops, causing a complex mechanical response of assembly [11,12,13,14]. However, how to identify contact loops and determine their edge number from complex contact networks has not been specifically described in relevant studies. For the assembly containing a large number of particles, the contact network has the characteristics of numerous nodes and complex loop shape, which requires a higher recognition accuracy of the contact loops. Based on the contact network, similar recognition methods of polygon loops include artificial recognition, classical image recognition techniques, and deep learning recognition methods (which developed rapidly in recent years) [15]. However, artificial recognition is inefficient and is not suitable for contact networks with a large number of loops. Classical image recognition techniques have been developed and have high accuracy for images with simple and good semantic results, but their algorithms need to be improved according to the image characteristics. The deep learning recognition method can recognize polygon types, but it needs a lot of samples to be labeled and trained before it can be applied [16]. Furthermore, the recognition of single or small numbers of images of contact loops with complex geometric shapes is inefficient. Therefore, based on the characteristics of the contact network, a new recognition method needs to be proposed to recognize the contact loop and judge its geometric type.

To understand the evolution of contact loops, some studies have been carried out by the discrete element method (DEM) [7,8,17,18]. For example, the shear dilatancy in the biaxial compression test can be interpreted as the transformation of contact loops from the small-dense structure to the large-loose structure [7]. However, the evolution representation of contact loops is often qualitative and lacks quantitative representation, especially the quantitative relationship between the structural indexes of contact loops at mesoscale and the mechanical indexes of assembly at the macroscale. The purpose of this study is to put forward a determination technique of contact loops with different geometric shapes and to establish the quantitative relationship between structural indexes and mechanical indexes. The structure of the paper is as follows. In Section 2, the recognition and determination technique for contact loops based on contact network images is introduced. Section 3 analyzes the evolution of meso-structural indexes in the biaxial compression test based on the above techniques. In Section 4, the quantitative relationship between meso-structural indexes and macro-mechanical indexes was established, and thus the bridge between meso-structure and macro-mechanics was also established. Finally, the conclusion and prospect of future work are given in Section 5.

## 2. Contact Loop Recognition and Determination Technique

**Figure 1:**
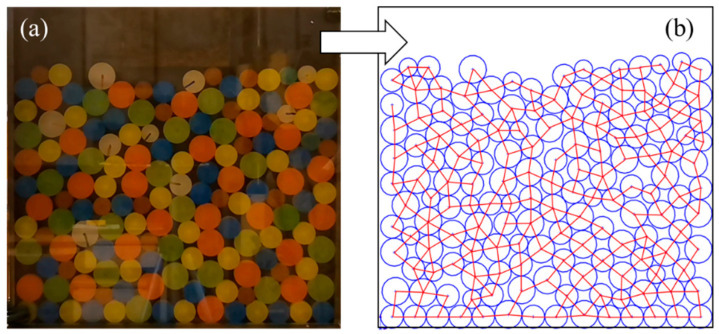
(**a**) Particle distribution image and (**b**) contact network image in the idealized 2D experiments (lamellar particles distributed in monolayer representing the 2D particle assembly).

For the DEM simulation with contact data, the contact network can be discretized into sub-domains (polygonal loops) by the “Delaunay triangulation” method and other analytical methods [7,17]. However, these methods are not suitable for cases without original contact data, such as contact network images obtained by 2D simulations and experiments (Figure 1). It is necessary to propose a 2D contact loop recognition and determination technique from the perspective of contact network images, which lacks original contact data. Therefore, one of the purposes of this study is to recognize and determine contact loops in contact network images without contact data.

There are three main reasons for developing the 2D recognition and determination technique. Firstly, contact forces in the 3D contact network are nonplanar. Such hierarchical relationships are difficult to represent in the image, which is a great challenge for the segmentation and identification of contact loops. The 2D technique in this study is an important basis for extending to future 3D researches. Secondly, much of the literature has demonstrated that 2D DEM simulations and idealized 2D experiments (such as Figure 1b) can reproduce the main mechanical features of mixed granular matters [19,20,21]. More importantly, the 2D model allows one to explore the microstructures in an effective but easier way than the 3D model [22]. For the evolution of the contact network, the effect of the 2D model is more intuitive. Therefore, the 2D recognition and determination technique is developed to investigate the meso-structure evolution of granular matters.

The principle of the 2D contact loop recognition and determination technique is that the corner number in the polygonal loop equals the side number of these polygonal loops. Therefore, the core of the technology is the recognition of corners. The existing corner detection technologies are mainly divided into gradient-based, template-based, and template-based gradient combinations. These algorithms include the *FAST* algorithm [23], *HARRIS* algorithm [24], *SUSAN* algorithm [25], and other improvement methods for corner detection. Based on the contact network image, we tested and compared the results of the above three corner detection algorithms.

**Figure 2:**
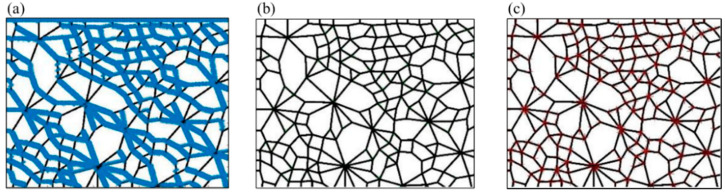
The corner recognition results of (**a**) *FAST* algorithm, (**b**) *HARRIS* algorithm, and (**c**) *SUSAN* algorithm.

In Figure 2, the *FAST* algorithm and the *SUSAN* algorithm recognize the points on the boundary as corners, resulting in a huge increase in the number of corners. However, the recognition effect of the *HARRIS* algorithm is the opposite and results in the loss of many corners. The traditional corner detection algorithm cannot obtain accurate corner information in the contact network image. Therefore, this study proposes a *Q-Y* algorithm to identify corners and then determine the geometric types of contact loops. The process of the recognition and determination technique based on the *Q-Y* algorithm is as follows.

**Figure 3:**
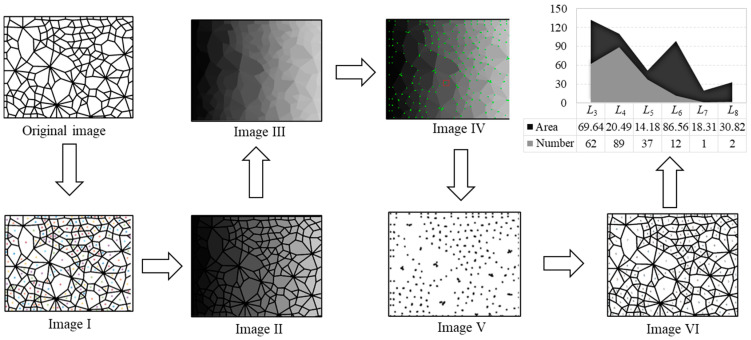
The specific process of the recognition and determination technique.

Firstly, we segment the original contact network image and get separate connected domains in Image I, wherein each connected domain represents a contact loop; secondly, we assign different gray value to each connected domain and obtain Image II; thirdly, we perform the morphological opening operation to integrate the divided edges of the two adjacent connection domains to obtain an edge to form Image III; fourthly, we use the *Q-Y* corner detection algorithm to perform corner detection on Image III to determine each corner point and obtain Image IV; fifthly, we set a gray value uniformly for the adjacent corner points as an intersection, and extract Image V containing only the intersection point; sixthly, we perform morphological corrosion processing on each intersection of Image V, and each intersection intrudes into the adjacent connection domain to obtain Image VI; seventhly, the geometric type of each connection domain is determined according to the principle that the number of sides is equal to the number of corners. The area of the connected domains is calculated by the number of pixels in each connected domain, and the statistical results (number and area of each geometric type) are obtained in the final step. The above process is demonstrated in Figure 3.

### 2.1. Contact Network Image Pre-Processing

The first step of the recognition and determination technique is image segmentation. That is, each contact loop is separated from the other in the contact network image. Each separated contact ring is regarded as a connected domain. The segmentation method based on the *OTSU* algorithm [26] has always been regarded as the optimal method for automatic image segmentation. The basic idea of this algorithm is to divide image pixels into two groups by a threshold, and then determine the optimal threshold by the maximum interclass variance between the pixels of two groups.

Suppose the grey levels of the contact network image is *G* = [0, *L* − 1] and the probability of each grey level is $P_{i}$. The threshold *t* divides the image into two groups *G*_0_ = [0, *t*] and *G*_1_ = [*t* + 1, *L* − 1]. The probabilities of the two groups are

(1) $$\{\begin{array}{c} \alpha_{0}=\sum_{i=0}^{t}P_{i}\\ \alpha_{1}=1-\alpha_{0} \end{array}$$

(2) $$\{\begin{array}{c} \mu_{0}=\sum_{i=0}^{t}\frac{iP_{i}}{\alpha_{0}}=\frac{\mu_{0}^{E}}{\alpha_{0}}\\ \mu_{1}=\sum_{i=i+1}^{L-1}\frac{iP_{i}}{\alpha_{1}}=\frac{\mu_{1}^{E}}{1-\alpha_{0}} \end{array}$$

where $\mu_{0}^{E}$ and $\mu_{1}^{E}$ are the expectations of *G*_0_ and *G*_1_, respectively; $\alpha_{0}$ and $\alpha_{1}$ are the probabilities of *G*_0_ and *G*_1_, respectively. Therefore, the interclass variance of the two groups can be expressed as

(3) $$\eta^{2}\left(t\right)=\alpha_{0}{\left(\mu -\mu_{0}\right)}^{2}+\alpha_{1}{\left(\mu -\mu_{1}\right)}^{2}=\alpha_{0}\alpha_{1}{\left(\mu_{0}-\mu_{1}\right)}^{2}$$

**Figure 4:**
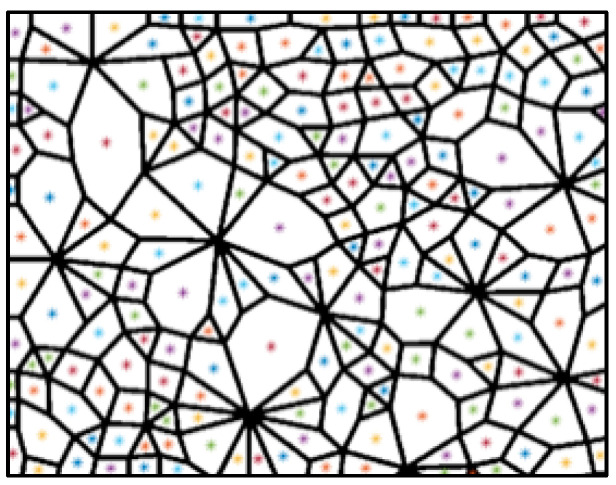
Segmentation result of the *OTSU* algorithm.

If $\eta^{2}\left(t^{*}\right)=\max\left(\eta^{2}\left(t\right)\right)$, then $t^{*}$ is the optimal threshold. If the value $t^{*}$ is not unique, the average value of all $t^{*}$ is used as the optimal threshold. For the contact network image, the *OTSU* segmentation method gives a more satisfactory segmentation result, as shown in Figure 4.

In the segmented image, different grayscales are assigned to the segmented connected domains. Since the existence of boundary lines of connected domains affects the effect of corner detection, the image needs to be processed using the algorithm of binary open operation to remove the boundary lines. The binary open operation includes corrosion calculation and expansion calculation, which is a multiple-point pattern-based unconditional simulation algorithm using morphological image processing tools [27,28].

The corrosion calculation can cause a shrinkage erosion of the image’s boundaries and is used to eliminate the small and meaningless areas. The definition of the corrosion calculation is the probing of an image *B* with a probe *S* to find a region *E* inside the image [29], which can be expressed as

(4) $$E=B\Theta S=\left\{\left(x,y\right)|S_{xy}\subseteq B\right\}$$

The expansion calculation is a pairwise operation of the corrosion calculation, which can be used to fill certain voids in the target area as well as to eliminate small particles of noise contained in the target area. The expansion calculation can be expressed as

(5) $$E=B⊕S=\left\{\left(x,y\right)|S_{\left(x,y\right)}\subseteq B\neq \emptyset \right\}$$

**Figure 5:**
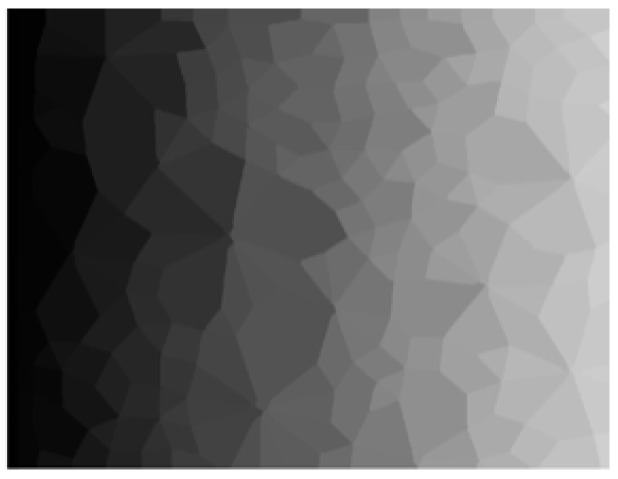
Greyscale assigned image without boundary lines.

The corrosion calculation can corrode boundary lines, and the expansion calculation can be used to fill the cavity in the target area and eliminate the noise contained in the target area. The processing effect of the binary open operation is shown in Figure 5.

### 2.2. Corner Detection

**Figure 6:**
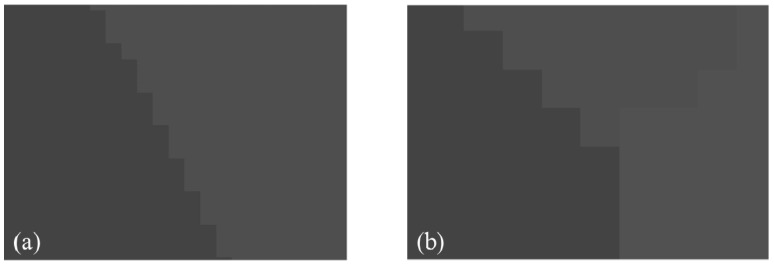
Grayscale distribution (**a**) around the boundary with two greyscales and (**b**) around the corner with three greyscales.

For the greyscale assigned image without boundary lines, there is a greyscale inside the connected domain, two greyscales around the boundary (Figure 6a), and three or more grey values around the corner (Figure 6b). The *Q-Y* algorithm for corner detection is proposed based on this characteristic.

If there are two or more grayscales within a certain range of the target pixel and they are different from its grayscale, the point is considered as a corner pixel. The specific process for corner pixel detection is as follows.

**Figure 7:**
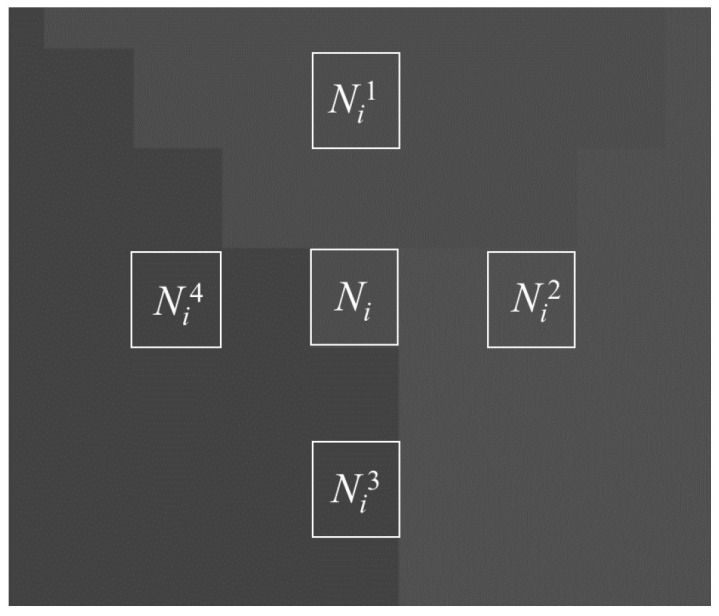
Spatial positions of *N_i_* and *N_i_^k^*.

Step 1: Four pixels $N_{i}^{k}$ around the target pixel $N_{i}$ are selected to check the grayscales, and the spatial positions of $N_{i}$ and $N_{i}^{k}$ are shown in Figure 7. If at least two pixels have different grayscale from the target pixel, then the target pixel may be a corner pixel and proceed to the next step; if not, then the next target pixel is checked.

Step 2: The corner response function is used to check whether the pixels $N_{i}$ screened in Step 1 are corner pixels. If the corner response function is satisfied, the pixel $N_{i}$ is regarded as a corner pixel. The corner response function is expressed as:

(6) $$\left\{ \begin{array}{l} N_{i}=g_{i}-g_{i}^{x}\\ N_{i\max}\neq N_{i\min}\neq 0 \end{array}\right.$$

where $g_{i}$ is the grayscale of $N_{i}$, and $g_{i}^{x}$ is the grayscale of the pixel $N_{i}^{x}$ around $N_{i}$.

**Figure 8:**
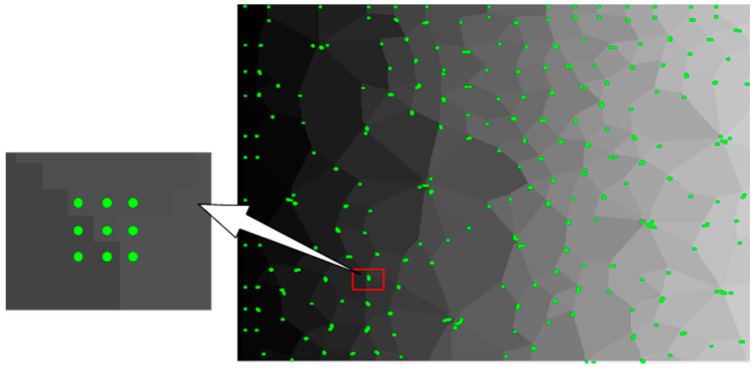
Recognition effect of the *Q-Y* algorithm.

Step 3: The pixels meeting the corner response function constitute corner pixels. It is worth noting that each corner may contain multiple corner pixels, as shown in Figure 8.

**Figure 9:**
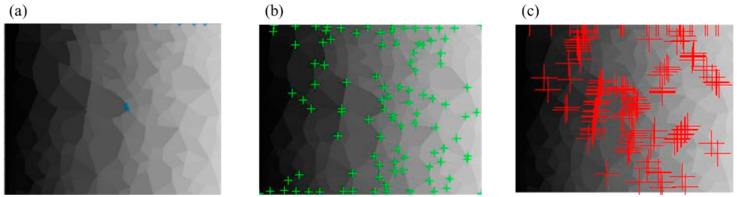
The corner recognition results of (**a**) *FAST* algorithm, (**b**) *HARRIS* algorithm, and (**c**) *SUSAN* algorithm for the greyscale assigned image without boundary lines (the bule, green, and red points are the recognized corners by the three algorithms respectively).

In Figure 8, the *Q-Y* algorithm identifies more than 99% of corners, which has a perfect recognition effect. For comparison, three common corner detection algorithms were selected again, whose results are shown in Figure 9. The *FAST* algorithm [23] identified only a few corners, the *HARRIS* algorithm [24] identified 80% of corners, and the *SUSAN* algorithm [25] identified corners including numerous non-corners and lost a large number of corners. The accuracy of these three algorithms is quite inaccurate compared to the *Q-Y* algorithm.

### 2.3. Recognition and Statistics

**Figure 10:**
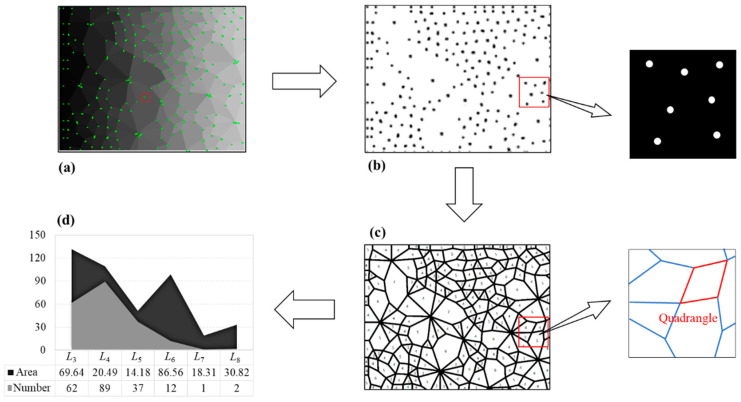
(**a**) Recognition results of corner pixels (the green points are the recognized corner pixels); (**b**) the adjacent corner pixels are considered as a corner; (**c**) the geometric type of contact loops is determined by the number of corners; and (**d**) the statistical results of the technique (number and area of each geometric type).

The adjacent corner pixels are assigned to the same value and considered as a corner. The above binary open operation affected the range of corner pixels, resulting in a decrease in the accuracy of corner allocation. Therefore, the corrosion calculation is required for Figure 8 so that every corner point can be allocated to the surrounding connected domains. After all of the corners are allocated, the number of corners in all connected domains can be obtained. Based on the principle that the number of corners in the connected domain equals the number of its sides, the geometric types of contact loops can be determined. Additionally, the area of a contact loop is represented by the number of pixels. Finally, the number and area of contact loops of each geometric type can be calculated. The above process can be represented in Figure 10.

The 2D contact loop recognition and determination technique can accurately determine and count the geometric types and their areas of contact loops, which creates conditions for quantitative analysis of contact network images.

## 3. Simulation and Analysis

The biaxial compression test was taken as an example and obtains the force chain images through the DEM simulation in this section. Based on the recognition and determination technique introduced in Section 2, the meso-structural indexes were calculated, and the relationships between the meso-structural indexes and the macro-mechanical indexes are analyzed.

### 3.1. Biaxial Compression Numerical Model

**Figure 11:**
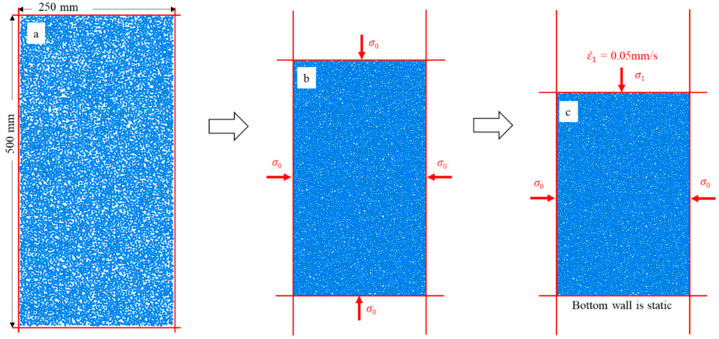
(**a**) Particles within the initial rectangular area; (**b**) the stress state for generating the dense assembly; and (**c**) the stress state for the biaxial compression test.

Deluzarche and Cambou [30] indicated that volumetric contracting strains are difficult to obtain in 2D and suggested that 2D simulations should be restricted to dense materials. To better reflect the strain responses, the dense assembly is adopted to match this suggestion. The dense assembly is produced by isotropic compression. First, an initial rectangular area consisting of four rigid walls was established (Figure 11a), which contained 10,000 round particles with uniformly distributed size (the minimum and maximum diameters of particles are 0.8 mm and 1.2 mm, respectively). Then, particles in the initial rectangular area were compressed isotropically. The servo control mechanism was used to continuously adjust the positions of the rigid walls until a stable confining pressure (*σ*_0_ = 20 kPa) was attained (Figure 11b). When the ratio of the mean unbalanced force to the mean contact force was less than 10^−5^, the assembly was considered to reach equilibrium, and a dense assembly was obtained.

After the dense assembly was generated, the biaxial compression was used to apply axial load. Biaxial compression means that confining pressures *σ*_0_ on the left and right walls remain constant by the servo control mechanism, and the axial load is applied by the downward movement of the upper wall (Figure 11c). The stress on the upper wall is the principal stress, which is denoted by *σ*_1_. In this study, the moving rate of the upper wall was maintained at 0.05 m/s. When the axial strain reaches 20%, it is considered that the particle has compression failure, and the loading stops.

The biaxial compression simulations are conducted using the DEM program PFC^2D^ code [31]. The linear elastic contact model was used to describe the contact behavior between particles, whose parameters were obtained according to previous experiments and numerical simulations. Specifically, the interparticle friction coefficient (*μ*_p_) is obtained by consulting previous DEM simulations, and the wall-particle friction coefficient (*μ*_w_) is set as 0 to ensure that the particles around walls can roll and slide without resistance. The value of normal-to-tangential stiffness ratio (*k_n_/k_s_*) is adopted as 4/3, which belongs to the range of 1.0 to 1.5 of realistic granular materials [32]. Damping constant *β* = 0.7, as suggested in Itasca [31], has been used for effectively dissipating the kinetic energy. The values of the contact parameters are shown in Table 1.

### 3.2. Number Evolution of Loops

**Figure 12:**
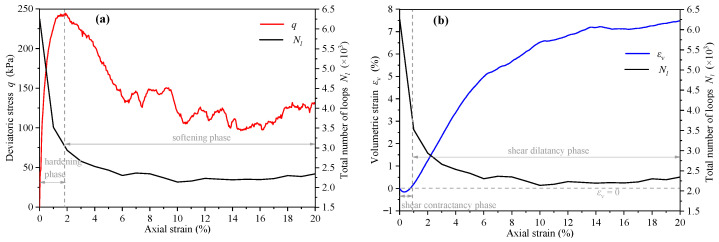
The variation curves of (**a**) loop number, deviatoric stress, and (**b**) volumetric strain with axial strain.

The variation of loop number *N_l_*, deviatoric stress *q* (*q* = *σ*_1_ − *σ*_0_), and volumetric strain $\varepsilon_{v}$ with axial strain $\varepsilon_{a}$ are shown in Figure 12. The deviatoric stress curve can be divided into the strain hardening stage and the strain-softening stage (Figure 12a). The curve of the loop number first decreases rapidly and then tends to be stable, and the inflection point appears with the peak stress, indicating that the change of contact loop is most active in the strain hardening stage. Figure 12b shows the assembly experienced the shear contraction stage ($\varepsilon_{v}\leq 0$) and the shear dilation stage ($\varepsilon_{v}>0$). The change of loops in the shear contraction stage was more active than that in the shear dilation stage.

**Figure 13:**
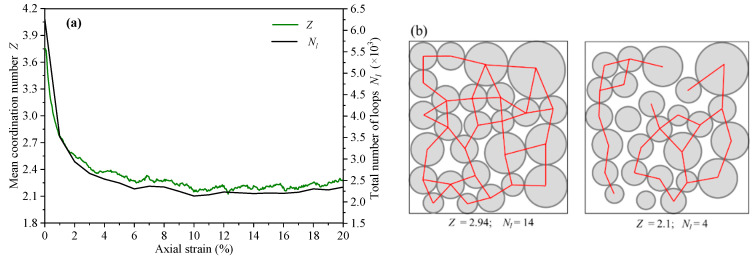
(**a**) The variation curves of loop number and average coordination number with axial strain; (**b**) the diagrams of the relationship between *Z* and *N_l_* for two examples.

In addition, the change of average coordination number *Z* was also explored. The evolution of *N_l_* is highly similar to that of *Z* (Figure 13a). This phenomenon can be explained by Euler’s relation of 2D topology. In the particle system, the relationship between the particle number *N*_p_, the contact number *N*_c_, and the loop number *N_l_* can be expressed as $N_{p}+N_{l}≅N_{c}$ [33]. Based on $Z=2N_{c}/N_{p}$, the relationship between *Z* and *N_l_* can be expressed as $N_{l}≅N_{p}\left(Z/2-1\right)$. For the assembly with a constant particles number, the average coordination number is positively correlated with the loop number. In order to show the relationship between the average coordination number and the loop number better, we drew two diagrams and calculated the corresponding values of *Z* and *N_l_*, as shown in Figure 13b.

### 3.3. Evolution of the Meso-Structural Indexes of Loops

The contact network is a link between the strength and deformation of granular matters. The number and structure of contact loops are highly correlated with the strength of the material, which can be used as a medium for carrying contact forces. Additionally, the macroscopic deformation can be explained in terms of the evolution of contact loops, considering the local contact particles in a loop-like mosaic. The evolution of the contact loops consists of the evolution of the number and area of loops with different geometrical types, which can be used to characterize the changes of the whole contact network. Therefore, the number percentage and the area percentage of the contact loops with different geometrical types are defined as the meso-structural indexes in this study.

**Figure 14:**
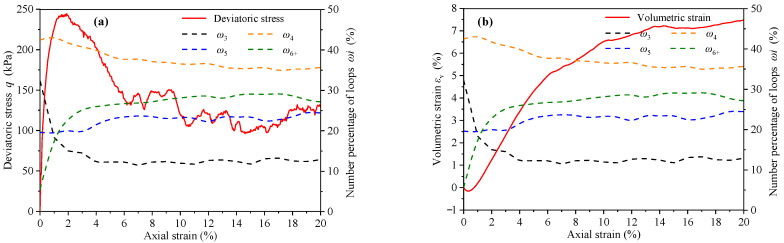
The variation curves of (**a**) deviatoric stress, (**b**) volumetric strain, and number percentage indexes (*ω*_3_, *ω*_4_, *ω*_5_, and *ω*_6+_) with axial strain.

$L_{i}$ denotes the contact loop with side number *i* (*i* = 3, 4, 5, …). The number percentage of $L_{i}$ is $\omega_{i}=N_{i}/N_{l}$, where $N_{i}$ is the number of $L_{i}$. In this study, the loops with the side number ≥ 6 are grouped into one group based on previous studies [7], which is hereafter referred to as *L*_6+_. Therefore, *L*_3_, *L*_4_, *L*_5_, and *L*_6+_ are counted separately in this paper and their number percentage *ω*_3_, *ω*_4_, *ω*_5_, and *ω*_6+_ are calculated. The variation curves of deviatoric stress, volumetric strain, and number percentage indexes with axial strain are shown in Figure 14.

Before the peak stress, *ω*_3_ decreased significantly, while *ω*_6+_ showed an obvious opposite evolution. In the softening stage after the peak stress, the evolution rates of *ω*_3_ and *ω*_6+_ gradually stabilized. It is worth noting that the maximum curvature of *ω*_3_ and *ω*_6+_ coincide with the minimum volumetric strain. In other words, the evolution rates of *ω*_3_ and *ω*_6+_ have undergone tremendous changes in the conversion process of assembly from the dense structure to the loose structure. However, *ω*_4_ and *ω*_5_ are basically constants and are not affected by the evolution of deviatoric stress and volumetric strain.

**Figure 15:**
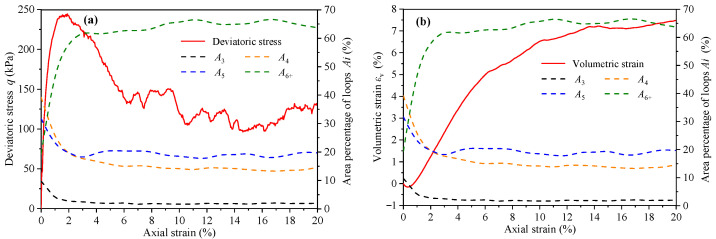
The variation curves of (**a**) deviatoric stress, (**b**) volumetric strain, and area percentage indexes (*A*_3_, *A*_4_, *A*_5_, and *A*_6+_) with axial strain.

The number percentage reflects the evolution of contact loops with different geometric types, but it cannot fully reflect the distribution degree of contact loops in the contact network. Therefore, it is necessary to introduce the area percentage of contact loops $A_{i}$, which means the area ratio of $L_{i}$ in the contact network. $A_{i}$ is defined as $A_{i}=S_{i}/S_{T}$, where $S_{i}$ and $S_{T}$ are the area of $L_{i}$ and the contact network, respectively. The variation curves of deviatoric stress, volumetric strain, and area percentage indexes (*A*_3_, *A*_4_, *A*_5_, and *A*_6+_) with axial strain are shown in Figure 15. The evolution of $A_{i}$ shows that only *L*_6+_ experiences an area increase while the other kinds of loop contract. In fact, having more sides gives rise to *L*_6+_ the ability to transform. The evolution of contact loops actually represents the evolution of assembly volume in the 2D simulation. The evolution trend of *A*_6+_ and $\varepsilon_{v}$ is similar, indicating that *L*_6+_ is the main factor affecting volume evolution.

## 4. Quantitative Analyses between Meso-Structure and Macro-Mechanics

Compared with the softening stage, the contact loops change more significantly in the hardening stage, which should be used as the focus of force chain evolution. The quantified analysis for the relationship between meso-structural and macro-mechanical indexes is established in the hardening stage ($\varepsilon_{a}\leq 2.0\%$).

In this section, the macro-mechanical indexes (deviatoric stress, axial strain, and volumetric strain) are used as dependent variables and meso-structural indexes (number percentage indexes and area percentage indexes) are used as independent variables to establish multivariate models. The independent variables could be reduced in dimensionality by the principal component analysis, and obtain the principal components [34,35]. Further, the multivariate models of the meso-structural and macro-mechanical indexes can be obtained by establishing a multivariate regression equation between the principal components and the independent variables.

### 4.1. Principal Component Analysis of the Meso-Structural Indexes

**Figure 16:**
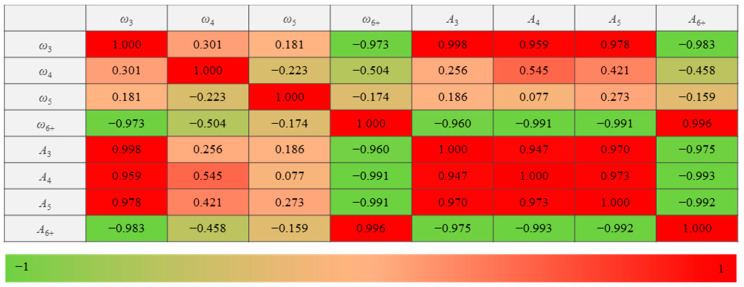
Multicollinearity analysis results among the eight independent variables.

As there are eight independent variables, multicollinearity may occur in this high-dimension analysis and compromise the statistical significance of independent variables. Multicollinearity occurs when the absolute value of the Pearson correlation coefficient is higher than 0.7 [36,37]. Pearson correlation coefficient (*R*) is defined as

(7) $$R=\frac{\sum_{i=1}^{n}(Y_{P}-\bar{Y_{P}})\cdot (Y_{A}-\bar{Y_{A}})}{\sqrt{\sum_{i=1}^{n}{\left(Y_{P}-\bar{Y_{P}}\right)}^{2}}\cdot \sqrt{\sum_{i=1}^{n}{\left(Y_{A}-\bar{Y_{A}}\right)}^{2}}}$$

where $Y_{P}$ is the predicted value, and $Y_{A}$ is the actual value. The statistical results of Pearson correlation coefficients among the eight independent variables are shown in Figure 16.

Figure 16 shows that the Pearson correlation coefficients of the diagonal can be higher than 0.7, indicating that multicollinearity can occur if all the variables are used. When multicollinearity occurs, the principal component analysis is suitable for the independent variables [38]. The principal component analysis is a multivariate statistical method that reduces multiple independent variables to a small number of principal components through dimensionality reduction techniques [39]. The principal components can reflect most information of the original variables and are linearly independent of each other. The eight meso-structural indexes in the hardening stage ($\varepsilon_{a}\leq 2.0\%$) are shown in Table 2.

The original data matrix X=n×p=21×8 was established from the data in Table 2, where *n* and *p* represent the number of samples and variables, respectively.

(8) $$X=\left[\begin{array}{cccc} x_{11} & x_{12} & \cdots & x_{n1}\\ x_{21} & x_{22} & \cdots & x_{n2}\\ \vdots & \vdots & \ddots & \vdots \\ x_{n1} & x_{n2} & \cdots & x_{np} \end{array}\right]$$

According to the definition of the overall principal component, the covariance of the principal component cov(F) is a diagonal array, which is expressed as

(9) $$\mathrm{cov}(F)=\left[\begin{array}{cccc} f_{11} & 0 & \cdots & 0\\ 0 & f_{22} & \cdots & 0\\ \vdots & \vdots & \ddots & \vdots \\ 0 & 0 & \cdots & f_{np} \end{array}\right]$$

The principal components *F*_1_, *F*_2_, …, *F*_p_ are uncorrelated with one another, which *F*_1_, *F*_2_, …, *F*_p_ are called first, second, …, *p*th principal components, respectively. The percentage of the variance of the *i* principal component $F_{i}$ in the total variance $f_{i}/\sum_{j=1}^{m}f_{j}(i=1,2,\ldots ,p)$ contribution rate is called the contribution rate of the principal component $F_{i}$. The contribution rate of the principal component reflects the ability of the principal component to synthesize the original variable information, and can also be understood as the ability to interpret the original variable [40]. The sum $\sum_{i=1}^{m}f_{i}/\sum_{j=1}^{m}f_{j}$ of the contribution of the first *m* (*m* ≤ *p*) principal components is called the cumulative contribution rate of the first *m* principal components, which reflects the ability of the first *m* principal components to explain the information of the original variables [41]. *X* is subjected to principal component analysis. According to the total variance explained table (Table 3), the percentages of the variance of the first three principal components are all greater than 10%, and the cumulative contribution rate has reached 99.868%, so it is sufficient to extract the first three principal components.

The extracted three principal components can remove implausible variables and determine the contribution of each variable to each principal component by using the component matrix. The component matrix is the coefficient of the factor expression of each variable, expressing the degree of influence of the extracted component on the meso-structural index. For the component matrix, the actual meaningful relationship between the components and the variables is not obvious. To make the coefficients more significant, the component matrix can be rotated so that the relationship between principal components and variables is redistributed and the correlation coefficients are differentiated towards 0 to 1. The relationship between principal components and meso-structural indexes can be derived from Table 3, and the rotated component matrix is shown in Table 4.

**Figure 17:**
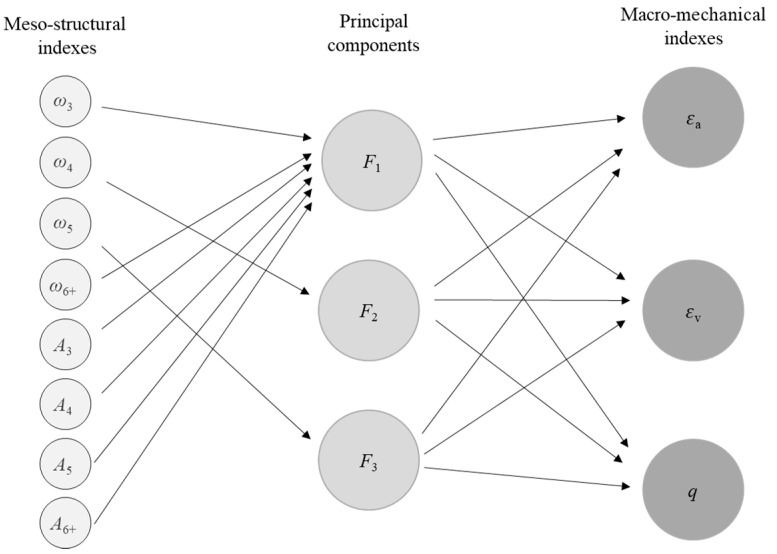
The relationship between meso-structural indexes, principal components, and macro-mechanical indexes.

By observing Table 4, it is found that each meso-structural index has a reasonable value of 1 (i.e., greater than 0.4), so none of the eight meso-structural indexes need to be deleted. The principal component *F*_1_, the highest percentage of contribution, is mainly influenced by *ω*_3_, *ω*_6+_, *A*_3_, *A*_4_, *A*_5_, and *A*_6+_ indexes, which can reflect the effect of the area percentages of all loops and the number percentages of *L*_3_ and *L*_6+_. The middle principal component *F*_2_ and the third principal component *F*_3_ are mainly influenced by *ω*_4_ and *ω*_5_, respectively, which reflects the effect of the number percentages of *L*_4_ and *L*_5_ is weak for the macro-mechanical indexes. Based on the rotated component matrix and the standardized coefficients (4.2), we can build the relationship between meso-structural indexes, principal components, and macro-mechanical indexes are shown in Figure 17, which reflects the contribution of meso-structural indexes to principal components and the effect of principal components to macro-mechanical indexes. Additionally, the influence degree between meso-structural indexes and principal components is quantized, showing as the component score coefficient matrix in Table 5.

The component score matrix indicates the relationship between each meso-structural index and each component, with a high score on a component indicating the closer the relationship between that indicator and that component. Based on the component score coefficient matrix, the functions and values of the three principal components *F*_1_, *F*_2_, and *F*_3_ can be obtained (Table 6) and used in place of the meso-structural indexes for the next step.

(10) $$F_{1}=0.233x_{\omega 3}-0.212x_{\omega 4}-0.12x_{\omega 5}-0.144x_{\omega 6+}+0.249x_{A3}+0.15x_{A4}+0.151x_{A5}-0.171x_{A6+}$$

(11) $$F_{2}=-0.207x_{\omega 3}+1.017x_{\omega 4}+0.163x_{\omega 5}-0.086x_{\omega 6+}-0.265x_{A3}+0.094x_{A4}+0.028x_{A5}-0.006x_{A6+}$$

(12) $$F_{3}=-0.09x_{\omega 3}+0.133x_{\omega 4}+1.027x_{\omega 5}-0.027x_{\omega 6+}-0.108x_{A3}-0.065x_{A4}+0.102x_{A5}+0.022x_{A6+}$$

### 4.2. Establishment of Multivariate Model Based on Principal Components

The feedback of meso-structural indexes on macro-mechanics was achieved by establishing multivariate models of the three principal components *F*_1_, *F*_2_, and *F*_3_ with axial strain $\varepsilon_{a}$, volumetric strain $\varepsilon_{v}$, and deviatoric stress *q*. Tolerance and variance inflation factor (VIF) was used to determine whether equations of the multivariate models were multicollinear, and the multivariate models were validated by variance analysis. The partial regression coefficients of the models were examined to determine the influence degree of the principal components on macro-mechanical indexes utilizing standardized coefficients [42].

The multivariate model between the axial strain $\varepsilon_{a}$ and the principal components *F*_1_, *F*_2_, and *F*_3_ is shown as

(13) $$\varepsilon_{a}=-0.505F_{1}-0.311F_{2}-0.104F_{3}+1$$

The variance analysis of the Equation (13) indicates an *F*-value of 89.912 with a *p*-value < 0.001, i.e., indicating that the multivariate model can be considered statistically significant at the *α* = 0.05 test level. Table 7 shows the results of the partial regression coefficient test. The *p*-values of all partial regression coefficients within the 95% confidence interval (95%CI) are less than 0.05, indicating that the significance levels of the partial regression coefficients are all in order. The standardized coefficients (*β*) for each principal component indicate that it can be seen that the principal component *F*_1_ has the greatest effect on the axial strain $\varepsilon_{a}$, and *F*_2_ and *F*_3_ have a small effect.

The multivariate model between the volumetric strain $\varepsilon_{v}$ and the principal components *F*_1_, *F*_2_, and *F*_3_ is shown as

(14) $$\varepsilon_{v}=-0.279F_{1}-0.31F_{2}-0.104F_{3}+0.341$$

The variance analysis of the Equation (14) indicates an *F*-value of 30.83 with a *p*-value < 0.001. Table 8 shows the results of the partial regression coefficient test. The *p*-values of all partial regression coefficients within the 95% confidence interval (95%CI) is less than 0.05. The standardized coefficient (*β*) for each principal component shows that the principal component *F*_2_ has the greatest effect on the volumetric strain $\varepsilon_{v}$, with the second-highest influence degree of the principal component *F*_1_, and they are about three times the influence degree of the principal component *F*_3_.

The multivariate model between the deviatoric stress *q* and principal components *F*_1_, *F*_2_, and *F*_3_ is shown as

(15) $$q=-68.249F_{1}-4.082F_{2}-3.992F_{3}+195.519$$

The variance analysis of the Equation (15) indicates an *F*-value of 753.49 with a *p*-value < 0.001. Table 9 shows the results of the partial regression coefficient test. The *p*-values of all partial regression coefficients within the 95% confidence interval (95%CI) are less than 0.05. The standardized coefficient (*β*) for each principal component shows that principal component *F*_1_ has the greatest influence on deviatoric stress *q*, being about five times more influential than principal component *F*_3_, and principal component *F*_2_ is more than three times the degree of influence of *F*_3_.

**Figure 18:**
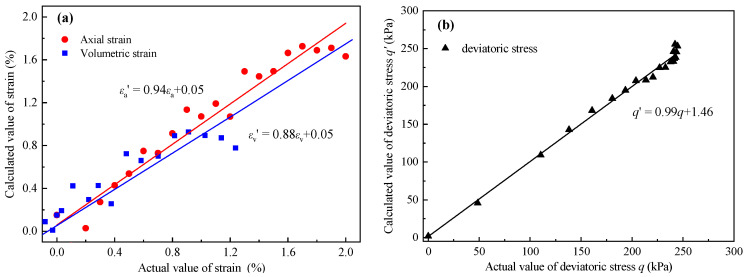
The fitting results between the calculated values and the actual values for (**a**) strain and (**b**) deviatoric stress.

For Equations (13)–(15), the Tolerance = 1 > 0.2 and the VIF = 1 < 10, demonstrating that there was no multicollinearity between the independent variables and no dimensionality reduction was required. Additionally, the actual values and the calculated values of three macro-mechanical indexes are fitted in Figure 18. For the fitting lines, the closer the slope is to 1, and the closer the intercept is to 0, the better the fit is.

The above analysis results show that the parameter estimation, hypothesis testing, and overall fit of the three multivariate models are good and statistically significant.

## 5. Conclusions and Outlook

A recognition and determination technique for 2D contact loops was proposed in this study. Taking the biaxial compression test as an example, the meso-structural indexes were calculated by the technique, and the relationships between the meso-structural indexes and the macro-mechanical indexes are analyzed. The main findings are summarized as follows:

(1) Based on the numerous and changeable polygonal loops in contact network images, the proposed *Q-Y* algorithm is effective in determining the geometric types of contact loops in contact network images.
(2) The change of contact loops is most active in the hardening stage, during which *ω*_3_ and *ω*_6+_ show opposite evolution patterns, while *ω*_4_ and *ω*_5_ are basically stable. The area evolution of contact loops represents the volume evolution of the 2D assembly and *L*_6+_ is the main factor affecting volume evolution.
(3) The variation of meso-structural indexes is active in the hardening stage, wherein the multivariate models between meso-structural indexes and macro-mechanical indexes were built.

The multivariate models in this study build a bridge between the mesoscale and macroscale of granular matters. Additionally, the contribution rate of meso-structural indexes to macroscopic mechanical indexes is quantified, which makes up for the deficiency of qualitative explanation only in existing studies. Although the multivariate models have a good verification effect, the multivariate models may have limitations due to the influence of some factors (such as stress condition and particle shape), since these factors may change the expression form of the multivariate models. Additionally, since the 2D recognition and determination technique is an image processing-based algorithm, it is difficult to extend the technique to 3D. Therefore, this study is limited to meso-structure and macro-mechanics of 2D DEM simulations.

Based on the techniques proposed in this study, it is suggested that the multivariate quantitative models can be further improved by changing the influences factors in the future in order to accurately feedback the macroscopic mechanical behavior of granular matter through the mesoscopic contact network. Of course, if the quantitative relationship between meso-structure and macro-mechanics can be verified experimentally, it would be of great significance for this area of study. 

## Figures and Tables

**Table 1:** Contact parameters used in the DEM simulations.

Parameters	Values
Interparticle friction coefficient, *μ*_p_	0.5
Wall-particle friction coefficient, *μ*_w_	0.0
Contact effective modulus, *E*_c_ (Pa)	1.0 × 10^8^
Normal-to-tangential stiffness ratio, *k*_n_/*k*_s_	4/3
Damping constant, *β*	0.7

**Table 2:** Mesostructural indexes in the hardening stage.

Axial Strain (%)	Meso-Structural Indexes							
	*ω*_3_	*ω*_4_	*ω*_5_	*ω*_6+_	*A*_3_	*A*_4_	*A*_5_	*A*_6+_
0	34.22%	40.80%	19.86%	5.13%	12.28%	38.49%	32.04%	17.19%
0.1	30.86%	44.14%	19.19%	5.80%	10.45%	40.43%	30.43%	18.69%
0.2	26.59%	45.89%	18.67%	8.85%	8.10%	37.76%	27.02%	27.13%
0.3	24.26%	45.21%	19.01%	11.52%	6.90%	33.49%	26.11%	33.50%
0.4	22.40%	44.57%	19.45%	13.58%	6.07%	30.85%	25.16%	37.92%
0.5	21.38%	44.62%	19.40%	14.61%	5.62%	29.12%	24.29%	40.97%
0.6	20.57%	44.81%	18.82%	15.79%	5.27%	28.03%	23.08%	43.63%
0.7	19.62%	44.49%	19.34%	16.55%	4.84%	26.90%	23.10%	45.17%
0.8	19.85%	43.39%	19.56%	17.20%	4.78%	25.35%	22.61%	47.26%
0.9	19.52%	43.23%	19.06%	18.19%	4.66%	24.80%	21.48%	49.06%
1.0	18.74%	43.00%	19.65%	18.60%	4.27%	23.77%	21.45%	50.51%
1.1	18.56%	42.76%	19.45%	19.22%	4.31%	23.34%	21.12%	51.24%
1.2	18.05%	42.60%	20.07%	19.28%	4.04%	22.77%	21.65%	51.53%
1.3	18.09%	42.32%	19.00%	20.60%	4.02%	22.26%	19.39%	54.32%
1.4	17.63%	42.62%	18.98%	20.77%	3.84%	22.22%	19.82%	54.12%
1.5	17.44%	42.15%	19.18%	21.23%	3.71%	21.52%	20.01%	54.75%
1.6	17.40%	42.07%	18.71%	21.82%	3.78%	21.38%	19.14%	55.70%
1.7	16.84%	41.81%	18.84%	22.51%	3.52%	20.85%	18.59%	57.04%
1.8	16.73%	42.29%	18.63%	22.36%	3.63%	21.18%	18.65%	56.54%
1.9	15.96%	41.37%	19.31%	23.37%	3.31%	20.40%	18.82%	57.47%
2.0	15.81%	41.71%	19.39%	23.10%	3.34%	20.08%	19.07%	57.51%

**Table 3:** Total variance explained of the first three principal components.

Component	Initial Eigenvalues			Rotation Sums of Squared Loadings		
	Total Variance	Percentage of Variance (%)	Cumulative Contribution Rate (%)	Total Variance	Percentage of Variance (%)	Cumulative Contribution Rate (%)
*F*_1_	6.123	76.534	76.534	5.692	71.156	71.156
*F*_2_	1.243	15.536	92.070	1.247	15.581	86.737
*F*_3_	0.624	7.797	99.868	1.050	13.130	99.868

**Table 4:** Rotated component matrix between the principal components and variables.

Variables	Principal Components		
	*F*_1_	*F*_2_	*F*_3_
*ω*_3_	0.994	-	-
*ω*_4_	-	0.960	-
*ω*_5_	-	-	0.985
*ω*_6+_	−0.949	-	-
*A*_3_	0.997	-	-
*A*_4_	0.942	-	-
*A*_5_	0.955	-	-
*A*_6+_	−0.967	-	-

**Table 5:** Component score coefficient matrix between meso-structural indexes and principal components.

Variables	Principal Components		
	*F*_1_	*F*_2_	*F*_3_
*ω*_3_	0.233	−0.207	−0.090
*ω*_4_	−0.212	1.017	0.133
*ω*_5_	−0.120	0.163	1.027
*ω*_6+_	−0.144	−0.086	−0.027
*A*_3_	0.249	−0.265	−0.108
*A*_4_	0.150	0.094	−0.065
*A*_5_	0.151	0.028	0.102
*A*_6+_	−0.171	−0.006	0.022

**Table 6:** Values of principal components under different axial strain.

Axial Strain/%	*F*_1_	*F*_2_	*F*_3_
0	2.92098	−2.36115	1.03283
0.1	2.2062	0.16253	−0.32189
0.2	1.25432	1.55719	−1.41124
0.3	0.72174	1.31898	−0.46048
0.4	0.29222	1.12591	0.71207
0.5	0.06105	1.18091	0.61629
0.6	−0.00741	1.11319	−0.88492
0.7	−0.27536	1.15016	0.50242
0.8	−0.27167	0.39496	0.98101
0.9	−0.22868	0.06347	−0.37464
1.0	−0.51139	0.19866	1.20855
1.1	−0.46837	−0.07702	0.66345
1.2	−0.68439	0.10974	2.32144
1.3	−0.48282	−0.5935	−0.60714
1.4	−0.55272	−0.33883	−0.58048
1.5	−0.59307	−0.5942	−0.09144
1.6	−0.49762	−0.87187	−1.35984
1.7	−0.62178	−0.9802	−1.03366
1.8	−0.60195	−0.71639	−1.56138
1.9	−0.79447	−1.06359	0.20003
2.0	−0.86479	−0.77897	0.44901

**Table 7:** Partial regression coefficient test results for Equation (13).

Principal Components	*β*	95%CI	*p*-Value
*F*_1_	−0.993	(−71.288, −65.210)	<0.001
*F*_2_	−0.059	(−7.121, −1.043)	0.011
*F*_3_	−0.058	(−7.032, −0.953)	0.013

**Table 8:** Partial regression coefficient test results for Equation (14).

Principal Components	*β*	95%CI	*p*-Value
*F*_1_	−0.596	(−0.373, −0.185)	<0.001
*F*_2_	−0.663	(−0.405, −0.216)	<0.001
*F*_3_	−0.223	(−0.199, −0.010)	0.032

**Table 9:** Partial regression coefficient test results for Equation (15).

Principal Components	*β*	95%CI	*p*-Value
*F*_1_	−0.814	(−0.582, −0.428)	<0.001
*F*_2_	−0.5	(−0.388, −0.233)	<0.001
*F*_3_	−0.167	(−0.181, −0.026)	0.012

## Data Availability

All the research data used in this manuscript will be available whenever requested.
